# The precision of value-based choices depends causally on fronto-parietal phase coupling

**DOI:** 10.1038/ncomms9090

**Published:** 2015-08-20

**Authors:** Rafael Polanía, Marius Moisa, Alexander Opitz, Marcus Grueschow, Christian C. Ruff

**Affiliations:** 1 Laboratory for Social and Neural Systems Research (SNS-Lab), Department of Economics, University of Zurich, 8006 Zurich, Switzerland; 2 Institute for Biomedical Engineering, University and ETH of Zurich, Zurich 8092, Switzerland; 3 Nathan Kline Institute for Psychiatric Research, Orangeburg, New York 10962, USA; 4 Center for the Developing Brain, Child Mind Institute, New York, New York 10022, USA

## Abstract

Which meal would you like today, chicken or pasta? For such value-based choices, organisms must flexibly integrate various types of sensory information about internal states and the environment to transform them into actions. Recent accounts suggest that these choice-relevant processes are mediated by information transfer between functionally specialized but spatially distributed brain regions in parietal and prefrontal cortex; however, it remains unclear whether such fronto-parietal communication is causally involved in guiding value-based choices. We find that transcranially inducing oscillatory desynchronization between the frontopolar and -parietal cortex leads to more inaccurate choices between food rewards while leaving closely matched perceptual decisions unaffected. Computational modelling shows that this exogenous manipulation leads to imprecise value assignments to the choice alternatives. Thus, our study demonstrates that accurate value-based decisions critically involve coherent rhythmic information transfer between fronto-parietal brain areas and establishes an experimental approach to non-invasively manipulate the precision of value-based choices in humans.

## Abstract (web summary)

Recent studies suggest that value-based choices involve communication between parietal and prefrontal cortices. Here the authors use a novel, non-invasive cortical manipulation technique to demonstrate a causal role for such communication in mediating accurate value-based, but not perceptual, choices.

## Graphical abstract

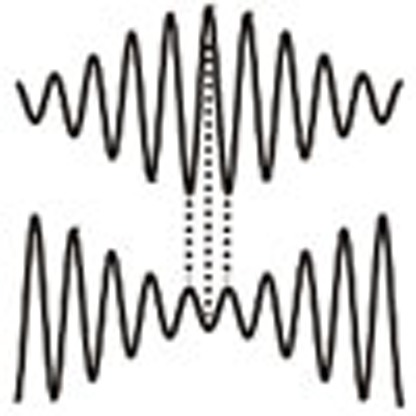

Taking choices based on the value of different options is fundamental for survival in most animal species, including humans^1^. A large body of research suggests that this cognitive function depends critically on integration of neural activity in several widely distributed brain regions^2^,^3^. For instance, to answer whether we want chicken or pasta, we must process the incoming sensory signals (for example, in terms of colour, shape, size, frequency and so on) to recognize the choice alternatives^3^,^4^, compute and compare the values for the different options based on sensory and mnemonic information^3^,^4^,^5^ and adequately map these value computations to the appropriate actions^6^.

Candidate brain areas that assign and compare values include the medial–prefrontal and parietal cortex, as demonstrated in single-unit recording studies in non-human primates^7^,^8^,^9^,^10^. However, it is largely unclear why value signals should be present in parallel for both of these regions. Recent theoretical models suggest that this may reflect an integrated circuit in which medial–prefrontal areas compute value signals^5^ and convey them to parietal areas, where these value signals are mapped to locations in space and/or appropriate actions^6^. The evidence for such accounts comes mainly from whole-brain imaging studies in humans, which infer such communication based on co-activation or functional connectivity between these areas^6^,^11^,^12^,^13^,^14^. However, due to the purely correlative nature of neuroimaging methods, it is so far unclear whether the statistical dependencies in fronto-parietal activity during value-based choices indeed reflect neural communication that is functionally relevant for the ability to take precise value-based choices, or just some functionally irrelevant by-products of the choice process. Moreover, most methods for the analysis of co-activations or connectivity do not allow inference on the directionality of this communication, so it is largely unresolved to what degree observed connectivity really reflects directed information transfer from medial frontal to parietal regions (but see ref. 6 for evidence based on fMRI consistent with that view).

One mechanism that may be specifically relevant for directed large-scale interactions is coherent phase-coupling of neural oscillations^2^,^15^, which is thought to lead to efficient impact of spiking in one neuronal population on interconnected populations. In line with that theory, a recent electroencephalography (EEG) study showed that the strength of phase coupling in neural oscillations of parietal and frontopolar regions predicted the accuracy of value-based choices^13^. However, several fundamental questions about the role of these large-scale interactions remain unresolved. For instance, is oscillatory synchronization between spatially distant brain regions—like the parietal and frontal cortices—indeed causally relevant for value-based choices, or is it just an epiphenomenon? Can this issue be investigated in healthy humans? If this neurobiological mechanism mediates precise value-based choices, then is it domain-specific or is it generally required for all types of decisions (for example, does it similarly influence value-based and perceptual choices)?

In the present work, we address these issues with the combination of a recently developed choice paradigm measuring perceptual- and value-based decisions with identical stimuli and motor responses^13^ together with a novel non-invasive stimulation technique that allows exogenous modulation of phase coupling between segregated cortical regions in healthy humans^16^,^17^ and a computational model of decision making that can disentangle distinct latent variables that correspond to different aspects of the decision process^18^. This allows us to test whether specific patterns of oscillatory coherence between frontal and parietal cortex are indeed functionally relevant for value-based choices.

## Results

**Figure 1:**
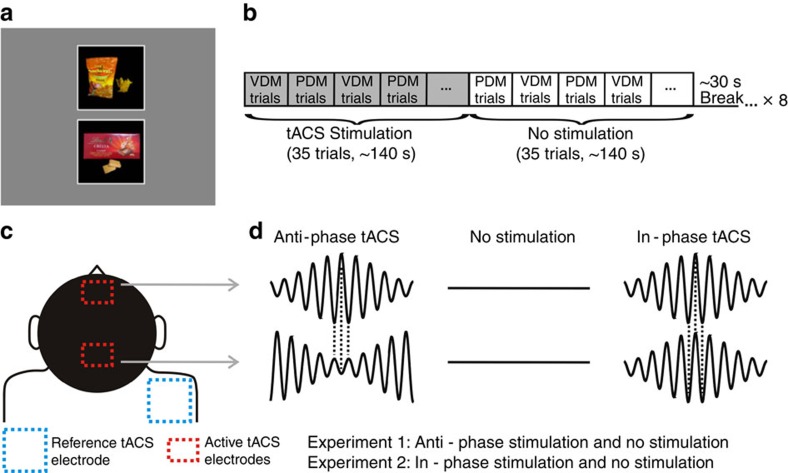
Behavioural paradigm and transcranial stimulation protocol. (**a**) Example screen from the decision stage. Participants were cued in advance about the type of decision that was required. For value-based decisions (VDM), subjects chose which item (the upper or the lower item) they preferred to eat at the end of the experiment. For perceptual decisions (PDM), they chose which item covered more of the black background. (**b**) Subjects alternated between blocks of PDM or VDM trials (6–10 trials per task-block). The order of the blocks was randomized across participants. Every 35 trials the stimulation was switched on or off. (**c**) tACS electrode set-up. Following the results of a previous EEG study^13^, the active electrodes were located over the mFPC (Fpz 10–20 EEG coordinate) and over the parietal cortex (Pz 10–20 EEG coordinate). A common and much larger reference electrode was mounted over the right shoulder. (**d**) Schematic of the oscillatory currents applied on each of the active electrodes. In experiment 1, subjects received either anti-phase stimulation (signals shifted by 180°) or no stimulation. Experiment 2 was identical to experiment 1, with the difference that subjects received in-phase stimulation (signals aligned at 0° difference) instead of anti-phase stimulation.

Our participants alternated between making perceptual or value-based choices on selected pairs of food stimuli^13^ (Fig. 1a,b, Methods). Perceptual choices required participants to choose the larger item, whereas value-based choices required them to pick the item they preferred to eat. For both types of decisions, stimulus pairs were identical but were preselected based on the participants' ratings to provide one of four different levels of evidence informing the perceptual or value-based choice (Methods). Perceptual evidence was defined as the absolute size difference between the stimuli, whereas value evidence reflected each pair's absolute value difference (in both cases, larger differences provide more evidence for the preferred choice option). The only difference between the two types of decisions was therefore which type of evidence needed to be accumulated for the choice; we could similarly define choice accuracy for both types of decisions as the consistency of decision outcomes with the previously acquired ratings of both stimuli within the choice pair^13^. During the decision period, the subjects alternated between blocks of perceptual or value-based choices (6–10 trials per task-block; see Methods for more details).

During the decision task, participants received transcranial alternating current stimulation (tACS), a non-invasive stimulation technique that entrains cortical rhythms in a frequency-specific manner^17^,^19^,^20^,^21^,^22^,^23^,^24^. We applied tACS in a multielectrode set-up shown to induce coupling or decoupling of behaviourally relevant neural oscillations between distant cortical areas^16^. We adapted this methodology to exogenously modulate the fronto-parietal oscillatory circuit (in the gamma band ∼55 Hz) that was previously shown to selectively correlate with the accuracy of value-based choices, but not with perceptual choice accuracy, general task performance or attention^13^. For this purpose, we placed two active electrodes at the specific scalp positions for which oscillatory coherence was observed in that study, located over medial frontopolar cortex (mFPC) and parietal cortex (Fig. 1c, Methods, and Supplementary Fig. 1). To confirm the electrode placement, we also computed the electric field distributions on the cortex resulting from our tACS electrode montage using a realistic finite element head model^25^. The maxima of the predicted electric field were relatively focal and occurred precisely in our regions of interest: the posterior parietal lobule and the medial frontopolar cortex (Supplementary Fig. 1). These were the very regions we intended to target with our manipulation, based on our scalp EEG data and previous MEG studies using a similar modelling approach during value-based choices^26^.

### Experiment 1

Healthy volunteers (*n*=27) received oscillatory currents at 55 Hz that were modulated by a 6 Hz envelope, to closely mimic the endogenous phase-amplitude modulation occurring in the human cortex during cognitive tasks^27^,^28^. Crucially, in this experiment the tACS oscillations were shifted by 180° (anti-phase condition, Fig. 1d) between both active electrodes. We hypothesized that this exogenous induction of anti-phase coupling would decrease the probability that presynaptic activity in one area would drive postsynaptic spikes in the second area^2^,^29^, thereby reducing neural coherence and the fronto-parietal information transfer thought to be relevant for the precision of value-based (but not perceptual) choices^13^.

**Figure 2:**
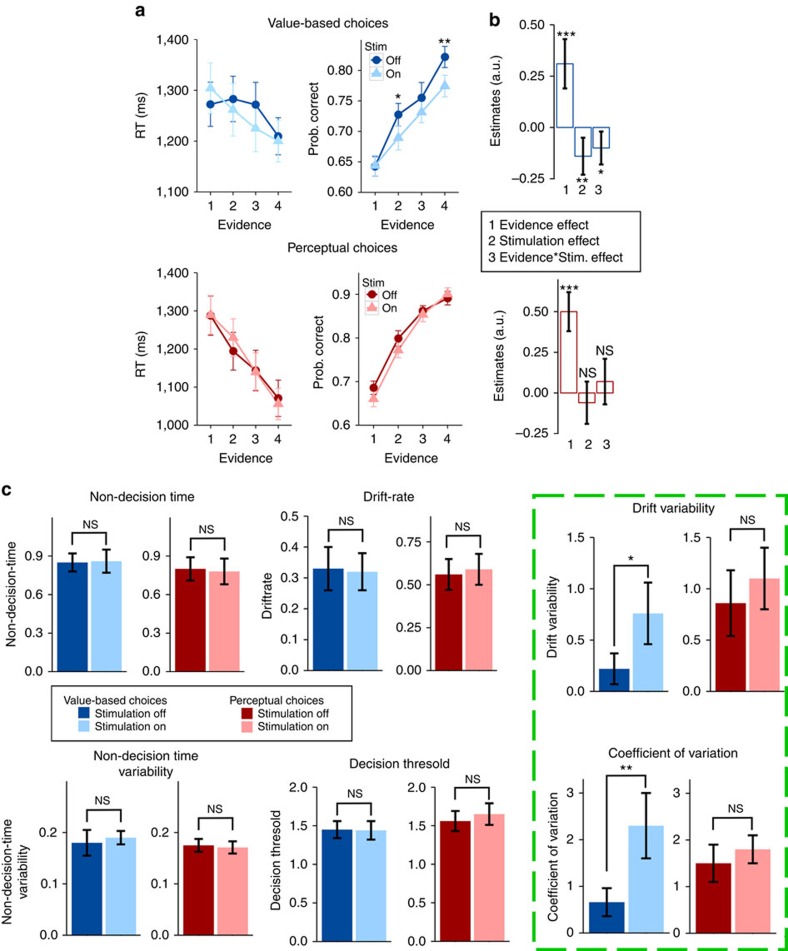
Anti-phasic tACS (experiment 1) decreases the accuracy of value-based choices and selectively increases value-based variability measured by DDM fits. (**a**) Observed mean reaction times including both correct and incorrect trials (left) and accuracies (right) for different levels of evidence in value-based (up) and perceptual choices (down) during off (light colours) and on (dark colours) stimulation periods. Error bars in this panel represent s.e.m. (**b**) Parameters from a multiple logistic regression of choice accuracy (*n*=27) on various regressors (see Methods). As expected, stronger evidence leads to more accurate choices for both types of decisions. Importantly, anti-phasic tACS significantly decreases the accuracy of value-based choices (negative main effect of tACS), and this effect scales up with the degree of evidence for one item over another (there is a significant interaction stimulation × evidence). Accuracies of perceptual choices remain unaffected. (**c**) Anti-phasic tACS selectively increases the trial-to-trial drift-rate variability parameter in value-based choices only (difference between the estimated posterior population distributions (Methods); see most right panel highlighted by the green dashed lines). All other parameters of the DDM remain unaffected by the stimulation. The main effect observed in the actual drift-rate between perceptual and value-based trials is caused by the fact that accuracies and RTs in perceptual trials were higher and faster, respectively. Error bars in **b** and **c** represent the 95% confidence interval range of the estimated effect sizes (**b**) and posterior estimates of the DDM parameters (**c**). *P<0.05, **P<0.01, ***P<0.001.

In line with our hypothesis, we found that value-based choice accuracy was indeed decreased during trials of anti-phasic stimulation (main-effect stimulation *β*=−0.133±0.05, *P*=0.009) and that this effect scaled up with the degree of evidence for one item over another (interaction stimulation × evidence *β*=−0.1±0.051, *P*=0.03; the largest effect was present for the highest evidence level (*T*(26)=3.22, *P*=0.003), whereas the smallest effect was observed for the lowest evidence level (*T*(26)=−0.05, *P*=0.95); see Fig. 2a,b). In contrast, perceptual choices were not affected by the tACS (main-effect stimulation *β*=−0.05±0.07, *P*=0.35; interaction stimulation × evidence *β*=0.07±0.069, *P*=0.3; Fig. 2a,b). A stimulation × evidence × task interaction confirmed the specificity of the tACS effects for value-based (but not perceptual) choices (*β*=0.11±0.043, *P*=0.02; Fig. 2a,b). None of these effects were influenced by the hunger level of the participants or by the other, choice-irrelevant type of evidence (Supplementary Table 1). Moreover, inspection of the data at the subject-level and non-parametric statistical analyses revealed that the effects of tACS were present in the vast majority of the subjects and were not due to the influence of outliers (Supplementary Fig. 2). Interestingly, the reduction in the accuracy of value-based choices was not accompanied by any significant increase in the reaction times (*P*>0.2 for all stimulation effects). Reaction times in the perceptual choices were virtually identical for periods with or without tACS (*P*>0.7 for all stimulation effects, Fig. 2a).

The results so far show that exogenous induction of desynchronization between mFPC and parietal regions specifically decreases the accuracy of value-based choices; however, these initial analyses do not provide insights into the mechanisms underlying the observed modulation of value-based choices. It is particularly unclear why stimulation had a significant effect on accuracies, but not on reaction times. To clarify these issues, we implemented the drift diffusion model^18^ (DDM), a well-established mathematical model of human choices that allowed us to disentangle how the manipulation of coherence affects several latent variables corresponding to distinct components of the decision process^30^. The latent variables we tested included the strength of evidence for the alternatives (mean drift rate parameter), the trial-to-trial reliability of the evidence (drift-rate variability), a decision caution parameter (threshold parameter), the time taken to initiate the choice and the motor responses (no-decision time), and its associated trial-to-trial variability (see Methods for details of the parameters used in this model; Supplementary Fig. 3). Fits of the model to empirical data fully reproduced the influence of tACS on choice behaviour (Supplementary Fig. 4). We found that during value-based decisions, tACS significantly increased the variability in the drift rate (see equation (5) in Methods; *P*_MCMC_=0.012, rightmost panel in Fig. 2c), while not affecting any other parameter of the DDM, including the mean drift rate (*P*_MCMC_>0.6, Fig. 2c) and the starting point variability (Supplementary Fig. 5). We also formally confirmed that the increase in drift-rate variability was indeed specific and not just due to any overall change in both drift rate and the associated noise, by analysing the coefficient of variation (a normalized noise-to-signal measure, defined here as the ratio of the drift-rate variability to the mean drift-rate). The coefficient of variation was indeed significantly lower during periods of anti-phasic tACS (*P*_MCMC_=0.008). These results strongly suggest that disruptions of fronto-parietal coherence by anti-phasic tACS did not affect the average quality of the value evidence *per se* (that is, the drift rate, (*P*_MCMC_=0.45, drift-rate panel in Fig. 2c) but rather reduced the precision with which the value-based evidence was represented from trial to trial. In other words, subjects became more variable (imprecise) from trial to trial in assigning values to the available choice alternatives, whereas the average value they assigned to the items across all trials, or the way that they generally accumulated the evidence, remained constant.

Importantly, our design ensured that the tACS effects unambiguously reflected influences on the actual choice process and not on the initial item rating period. This is because any possible noise in the initial item ratings was perfectly matched between the active and the sham stimulation, which were given in a within-subject design. Valuation noise was therefore factored out of the analysis of the effect of stimulation on choice.

The same stimulation protocol applied during perceptual trials left all of the DDM parameters unaffected (*P*_MCMC_>0.3 for all DDM parameters), thereby showing that the tACS selectively affected the precision of value representations. This latter conclusion was statistically confirmed by a significant stimulation × task interaction for the coefficient of variation (*P*_MCMC_=0.018). The same interaction was also observed for control analyses (*P*_MCMC_=0.032) of trials with matched behavioural performance, as implemented by comparing trials with selected evidence levels from the sham stimulation condition for perceptual (only evidence levels 1 and 2) and value-based choices (only evidence levels 2 and 4; this was confirmed by comparing the behavioural data between perceptual and value trials at the above-mentioned evidence levels using paired *t*-tests: *T*(26)=0.25, *P*=0.8 for reaction times (RTs) and *T*(26)=0.92, *P*=0.36 for accuracies). This demonstrates that the tACS-induced effects are indeed specifically related to the precision of value-based choices and cannot be explained by possible influences on attention or other task-general cognitive functions.

Even though we did not observe significant effects of the tACS on reaction times, one may argue that the stimulation induced a tendency for participants to speed up (Fig. 2a, top left). In the context of the DDM, faster reaction times accompanied by lower accuracy levels are often thought to result from changes in the decision boundary or the drift rate^31^,^32^, two parameters that were not affected here. Importantly, DDM simulations confirmed that the specific pattern we observed (slightly faster RTs accompanied by lower accuracies) can result from increased variability in the drift-rate without any change in decision boundary or mean drift rate (Supplementary Figs 7 and 8). The explanation for this is that higher variability in the drift rate increases the likelihood of the decision variable to reach a boundary (irrespective of whether this is correct or incorrect), resulting in faster responses and poorer choice accuracies.

The tACS mainly affected performance on trials with a high value difference between both options (interaction stimulation × evidence *β*=−0.1±0.051, *P*=0.03; Fig. 2a,b), which may appear somewhat counterintuitive if one assumes that disruptive stimulation should mainly affect trials with weak evidence representation. However, this specific interaction evident in our results is precisely predicted by the DDM fitted to our empirical data. Panel a in Supplementary Fig. 6 displays the fitted-DDM prediction that the maximum difference in accuracy between the two conditions caused by tACS should occur at intermediate levels of evidence (close to Evidence Level=4, the maximum level of evidence in our study, see Supplementary Fig. 6b). The DDM also reveals that this specific interaction of evidence level and stimulation is mediated by the change of drift-rate variability induced by the tACS (Supplementary Fig. 6c). This is evident in DDM simulations of systematic variations in drift-rate variability, which result in a monotonic decrease of accuracies as a function of increased drift-rate variability. The maxima of this decrease are again located at intermediate evidence levels (approximately at Evidence Level=4, Supplementary Fig. 6c), fully consistent with the effects we observe in our data. Therefore, the DDM can provide a mechanistic explanation for the somewhat counterintuitive results we observe, namely that the tACS-induced reduction in choice accuracy for intermediate (∼78%) levels of accuracy is caused by increases of the trial-to-trial variability in the readout of the value evidence.

### Experiment 2

**Figure 3:**
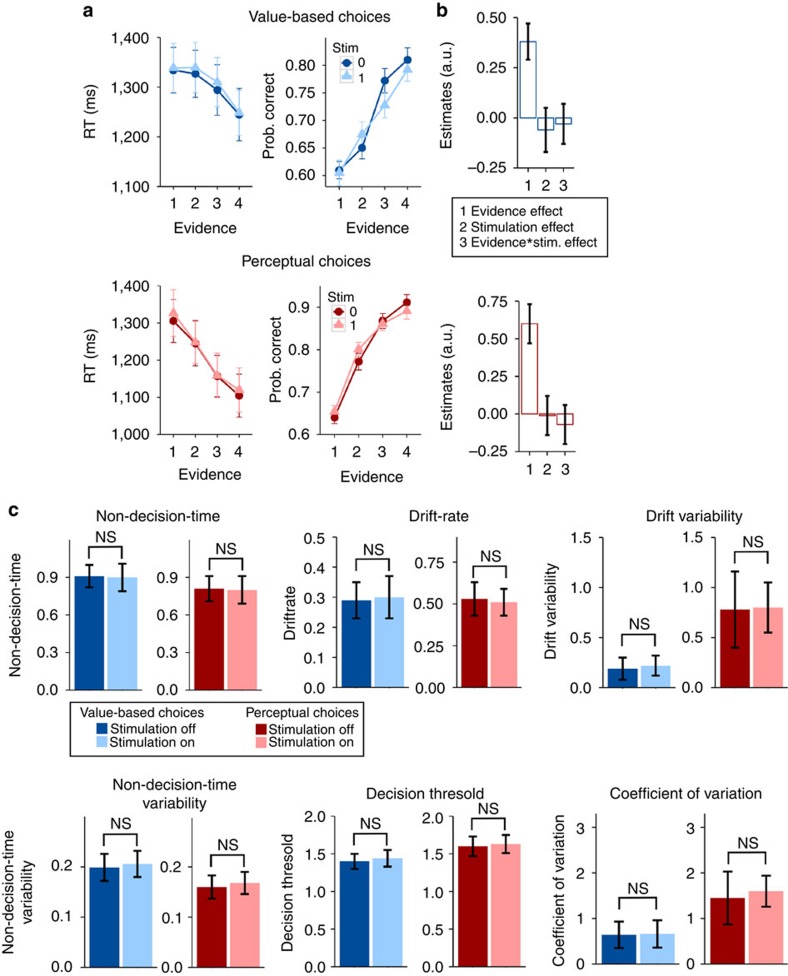
In-phase tACS has no effects on behavioural performance. In this figure the number of replicates, statistical tests and error bars are the same as Fig. 2, but this time for the in-phase tACS condition (experiment 2). The tACS protocol had no effect on behaviour or on any of the estimated model parameters.

In order to confirm that the results obtained in experiment 1 were specifically due to disruption of coherence between the stimulated sites, rather than any influences on the local activity in each of the areas, a new set of participants (*n*=27) took part in a second tACS protocol. The oscillations induced through the mFPC and parietal electrodes were identical with those used in the first experiment, but they were now perfectly aligned (that is, phase difference=0; Fig. 1d). Here we expected either a performance enhancement for value-based choices or no change in behaviour. We found that this stimulation protocol did not significantly change accuracies or reaction times for either tasks (*P*>0.2, for all factors including stimulation; Supplementary Table 3) and also had no influence on any of the DDM parameters (Fig. 3). We speculate that the absence of effects could reflect two possible explanations: first, synchronization of presynaptic spikes from a sending population must coincide with the presynaptic spikes in a receiving area in a precisely timed manner^2^,^29^. In our experiment we used a time lag of zero, but the optimal lag might be different from zero^29^ and somewhat variable across subjects^33^. Second, participants taking part in our study were young healthy volunteers; it is thus possible that mFPC–parietal phase coupling is so close to optimal that improvements in value-based choice performance are difficult to achieve with our zero-lag stimulation protocol. In this latter case, however, a tACS-related enhancement of performance may still be possible in individuals with neuropathologies associated with abnormal large-scale synchronization^34^. Irrespective of these considerations, our results from experiment 2 clearly show that the influence of our anti-phasic tACS protocol on the precision of value-based choices (experiment 1) is not due to an entrainment of neural oscillations or noise at either site alone, but specifically reflects the disruption of temporally precise phase coherence of mFPC–parietal oscillations.

### Experiment 3

In the previous experiments, we administered tACS to the two locations at either perfect counter-alignment (180° phase shift, experiment 1) or in full alignment (0° phase shift, experiment 2). However, as discussed above, it may be argued that these phase shifts in the exogenously applied protocols are not ideally suited to influence biological phase coupling between the two stimulated regions, which may have to account for the delay in neural transmission of signals between both sites. We therefore conducted a new experiment that allowed us to explicitly test for this delay, thereby confirming our results and mechanistically specifying the fronto-parietal phase coupling underlying value-based choices. To this end, a new set of participants (*n*=32) received tACS over the mFPC and parietal cortex at six different phase lags between the oscillations over the two sites (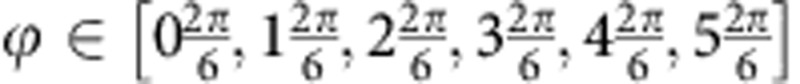 and sham stimulation condition). Importantly, all conditions were given in a within-subject design, within the same experimental session. In that session, we focused on value-based choices only, at an evidence level that closely matched the accuracy level observed for the easiest perceptual choices in experiments 1 and 2 (accuracy ≈87%). This allowed us to confirm that the tACS-induced effects apply only to value-based choices and do not relate to demands imposed by switching between the two tasks, or to focusing attention on one stimulus dimension while avoiding distraction of the non-relevant evidence (as it could have been the case in experiments 1 and 2).

**Figure 4:**
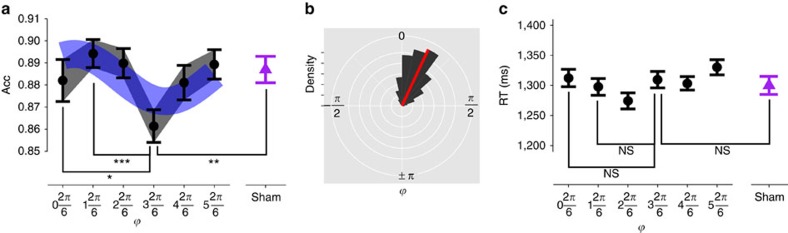
The accuracy of value-based choices is parametrically modulated by the phase of fronto-parietal gamma-band coupling. (**a**) This panel shows a sinusoidal parametric effect of the different tACS phase lags on the accuracy of value-based choices (*n*=32, mixed-effects regression). The *x* axis represents the phase difference between the tACS oscillatory activity in the frontopolar and the parietal electrodes (*ϕ*). Filled black dots represent choice accuracy for each stimulation condition and the purple triangle represents the accuracy in the sham condition. The blue area represents the 90% confidence interval for the prediction of the sinusoidal model fitted to the empirical data. Statistical comparisons show that full anti-phase stimulation 
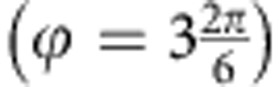
 is significantly different from full in-phase stimulation 
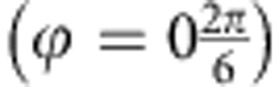
, sham (purple triangle), and also from the tACS condition with the optimal behavioural performance 
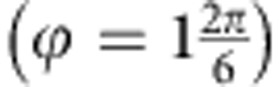
 (**b**) The sinusoidal model fitted to the empirical data (see panel a) shows that at the optimal synchronization level, the mFPC phase leads with respect to parietal phase by 24°±15° (red line, ± represents s.d. of the population estimate). (**c**) Reaction times were not systematically altered by the different-phase tACS conditions. Full anti-phase stimulation 
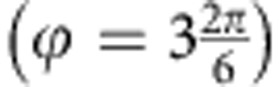
 was not different from full in-phase stimulation (that is,
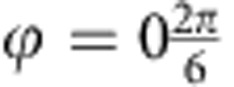
), sham, or the optimal tACS condition 
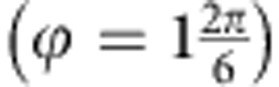
. *P<0.05, **P<0.01, ***P<0.001. All error bars depict s.e.m.

We reasoned that if choice behaviour is directly modulated by the phase of synchronized activity between the tACS protocols over both sites, then we should observe that choice accuracy should be better explained by a sinusoidal parametric model (as a function of the phase difference between the fronto-parietal tACS oscillations) than by a model with a single intercept. The sinusoidal model used here can be conveniently written as a linear function of sines and cosines, that is, accuracy=*β*_0_+*β*_1_ sin(*ϕ*)+*β*_2_ cos(*ϕ*) (see Methods for details). In line with our hypotheses, we found that the sinusoidal model explained the data better than the constant model or even a simple linear model (DIC_circular_=−668, DIC_constant_=−661, DIC_linear_=−660; note that the smaller the DIC the better the fit; Fig. 4a, see also Supplementary Table 3). Full anti-phase stimulation significantly reduced performance relative to both sham (planned *post hoc* comparisons; *T*(31)=2.5, *P*=0.008) and full in-phase stimulation (*T*(31)=1.9, *P*=0.03). The maximum accuracy occurred at 
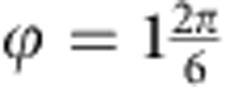
, with a highly significant difference to full anti-phase stimulation (*T*(31)=3.74, *P*<0.001). In line with experiment 2, we did not find a significant difference between sham and full in-phase tACS (*T*(31)=0.43, *P*=0.67). Moreover, we reproduced the finding from experiments 1 and 2 that reaction times were not affected by tACS (Fig. 4c and Supplementary Table 3). Thus, experiment 3 fully replicated the patterns of results obtained in experiments 1 and 2, but also demonstrated that the effects of tACS on performance are indeed specifically tied to the phase of the ongoing oscillations. Moreover, since accuracy levels in experiment 3 were similar to the accuracies for the easiest evidence levels in perceptual choices (experiments 1 and 2), we conducted a cross-over comparison between these experimental conditions. This analysis revealed a significant interaction (AntiPhase/sham) × (value-based/perceptual choices) (*P*_MCMC_<0.008). Thus, we could again confirm that the oscillatory coherence disrupted by tACS selectively relates to value-based choices and not to general cognitive functions such as attention or task switching.

The precise pattern of value-based choice accuracy across the different phase angles could also provide us with information on the directionality of the prefrontal–parietal interactions. Using trigonometric operations, we estimated the most likely oscillatory difference between the frontopolar and parietal cortex from the fitted circular model coefficients, by applying the following formula:


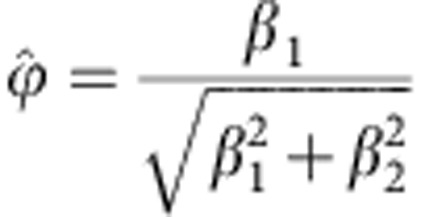


This revealed that the frontopolar phase most likely leads with respect to the parietal phase by 24°±15° (± represents s.d. of the population estimate; see Fig. 4b). Interestingly, this empirical result is in line with suggestions from recent fMRI effective connectivity studies^6^ that frontal regions may influence the activity of parietal regions during value-based choice evidence accumulation. Thus, by specifying the most plausible phase lag between the frontal oscillatory phase with respect to the parietal phase, our data provide empirical evidence for suggestions that computations of value signals taking place in medial–prefrontal and frontopolar regions^6^,^7^,^8^,^13^,^35^,^36^ may serve as inputs to the accumulation of perceptual and/or action-related evidence in parietal regions^6^,^11^,^37^,^38^.

## Discussion

Our study shows that value-based choices not only involve specialized local computations in frontal and/or parietal areas (as has been shown in recent fMRI^39^, EEG^13^, MEG^26^ and single-unit recording^8^,^9^ studies) but are causally influenced by the degree of rhythmic synchronization between mFPC and parietal cortex. This synchronization specifically affects the precision of value-based but not perceptual choices, and this effect does not relate to differences in attentional function, task-switching requirements or general performance between both types of decisions. However, even though perceptual choices were not affected by the present manipulation, our results do not rule out that they may require other types of communication between parietal and frontal regions. In fact, there is compelling evidence that other regions of the prefrontal cortex—such as the inferior frontal junction and frontal eye fields—play a crucial role in top–down influences on sensory areas and parietal brain regions during perceptual choices^33^,^40^,^41^,^42^,^43^. Our study now demonstrates that oscillatory coherence between the mFPC and parietal sites is crucial for value-based choices, but not for matched perceptual decisions. This dissociation highlights fronto-parietal coherence as a general mechanism underlying different types of decision making, which may be expressed in different task-specific networks depending on the type of information needed for the choice. This hypothesis could easily be tested in future studies of other types of decisions using the non-invasive brain stimulation approach established here.

Our modelling results show that disruption of fronto-parietal coherence resulted in lowered precision of value-based food choices. In other words, on any given trial, participants' choices became more inconsistent with their preferences as stated before the experiment, but the average preference across trials remained stable. This specific change in the trial-by-trial variability of value-based choices appears fully consistent with recent proposals about the role of fronto-parietal information transfer in value-based choices. These accounts assume that medial–prefrontal and orbitofrontal regions locally integrate sensory and mnemonic information (from sensory cortices, the striatum and the hippocampus) to compute values for the different choice alternatives^4^,^5^,^7^,^8^. These value signals are then conveyed to parietal regions, where they can be mapped to specific locations in space and/or the appropriate motor actions. This process most likely occurs through functional interactions between medial–prefrontal and parietal regions^6^,^11^,^13^,^14^, possibly by means of direct anatomical connections between the orbitofrontal cortex and the intraparietal sulcus via the third branch of the superior longitudinal fasciculus (SLF-III)^44^. The tACS-induced variability in the choice process observed here appears consistent with the notion that the stimulation may have specifically disrupted the value-to-action transformations^6^ mediated by the fronto-parietal information transfer, while not differentially affecting the localized prefrontally mediated memory-based value computations *per se*.

Although the effects of tACS were statistically significant and reproduced by two independent experiments, they were rather slight in absolute terms. Does this indicate that fronto-parietal communication only has limited functional importance for value-based choices? We do not believe this is the case, for three reasons. First, non-invasive brain stimulation methods differ substantially from brain lesions in that they do not fully obliterate neural activity and the ability to implement the related behaviour—they usually only reduce and/or slow performance^16^,^17^, as also observed here. Second, our results are fully consistent with the strength of behavioural changes as predicted by model simulations (see Supplementary Fig. 6). In other words, if stimulation does not affect the overall strength of the evidence for one item over another, but only the reliability with which this is mapped to the alternatives, then the model predicts only slight absolute effects on performance, as indeed observed in our data. Third, even though our tACS protocol was fully motivated by the previous EEG results^13^, it is theoretically impossible to know whether the resulting parameter combination (for example, stimulation intensity, frequency, electrode positioning and so on) is indeed optimal for affecting value-based choices. The impact of tACS could therefore be optimized in future research by systematic changes to the protocol, for instance, by selecting tACS frequencies in a personalized manner or by investigating what electrode montages allow more focalized modulations in the cortical areas of interest.

Taken together, our findings demonstrate the functional relevance of oscillatory large-scale brain synchronization for value-based choices and they show a direct link between observed choice precision and communication through coherent oscillations. More generally, our study establishes an experimental approach to non-invasively manipulate the precision of value-based decisions in the human brain. This could be important for clinical and developmental neuroscience, as pathological variability of value-based decisions is a key symptom of various disorders (for example, addiction, obesity and other impulse control disorders^1^,^34^,^45^) associated with disrupted functional large-scale connectivity.

## Methods

### Participants and behavioural paradigm

Healthy right-handed volunteers (*n*=86, age 20–30 years, 30 females; *n*=27 in experiment 1, plus *n*=27 new participants in experiment 2, plus *n*=32 new participants in experiment 3) were included in the study. Subjects were informed about all aspects of the experiment and gave written informed consent. None of the participants suffered from any neurological or psychological disorder or took medication that interfered with participation in the transcranial stimulation study. Subjects received monetary compensation for their participation in the experiment, in addition to receiving one food item (see below). The experiments conformed to the Declaration of Helsinki and the experimental protocol was approved by the Ethics Committee of the Canton of Zurich.

For all experiments, subjects were asked not to eat or drink anything for 3 h before the start of the experiment. After the experiment, subjects were required to stay in the room with the experimenter while eating the food item that they chose in a randomly selected trial of the value-based decision making task (see below). All experiments took place between 0800 and 1700 hours. The experiment consisted of two main steps: (1) the rating phase, and (2) the decision-making task. In the rating phase, we asked the participants to provide subjective perceptual- and value-based ratings about the same set of 65 food images using an on-screen slider scale. All of the food items were in stock in our lab and subjects were notified about this. For value-based ratings, participants indicated ‘how much they wanted to eat the presented food snack at the end of the experiment' (scale from −10 to 10). For perceptual ratings, we asked the participants to provide an estimate of ‘how much (in per cent) they thought the food item was covering the black background within the white square' on a scale from 5 to 100% in steps of 5% (ref. 13). Before providing the ratings, subjects briefly saw all of the items for an effective use of the value-based rating scale. Similar to our previous study^13^, the ratings were well distributed across the rating scale.

Immediately after the ratings, an algorithm selected a balanced set of perceptual and value-based trials divided into four different evidence levels based on the individual subjective ratings provided by each participant. Evidence levels for the value-based task were:


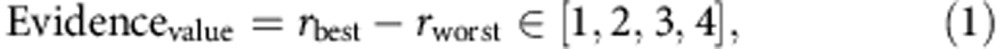


and for perceptual trials:


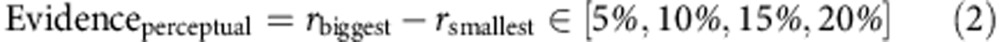


Where *r* represents the initial rating, and *r*_best_−*r*_worst_ and *r*_biggest_−*r*_smallest_ represent the difference of the ratings (that is, the evidence) for a given trial in value-based and perceptual choices, respectively.

Decision-making trials started with the central presentation (for 3 s) of a word (length ∼0.8°, height ∼0.3°) indicating whether subjects were in a perceptual trial (word ‘LIKE') or in a value-based trial (word ‘AREA'). On the subsequent screen, this task cue word was replaced by the letter ‘L' or ‘A' (∼0.2°) to remind subjects that they were in a value-based or perceptual block, respectively. Two food items were simultaneously displayed, one above and one below (*y* eccentricity 3.6°; a white square of 6° width surrounded each food item, see Fig. 1a). In the value-based trials, subjects indicated which item (upper or lower) they would prefer to consume at the end of the experiment, while in the perceptual trials, subjects indicated which item (upper or lower) covered more area within the white square. To make these choices, subjects pressed one of two buttons on a keypad with their right-index finger (upper item) or their right thumb (lower item). Subjects had 4 s to make a decision; otherwise the trial was marked as a ‘miss trial'. We defined a correct choice as a trial in which the subject chose the item with a higher rating from the separate rating tasks. Each experimental session consisted of 560 trials divided into 8 runs of 70 trials each. The maximum number of consecutive perceptual or value-based trials in a single block was pseudorandomized to be between 6 and 10 trials. The 560 trials were fully balanced across all factors (Trial type: perceptual or value-based; Evidence Level: 1, 2, 3 or 4; Correct response: Up or Down).

### Transcranial alternating current stimulation (tACS)

tACS was delivered through two current stimulators (NeuroConn) connected to a common reference. Following our previous EEG study^13^ (see also Supplementary Fig. 1), we placed two active electrodes (5 × 7 cm, transversally mounted) over the scalp locations where we had observed fronto-parietal coherence during value-based choices, one over the mFPC (Fpz 10–20 EEG coordinate) and the second over the parietal cortex (Fig. 1c, Pz 10–20 EEG coordinate). Electrodes were attached to the scalp with the Ten20 conductive paste (Weaver and company, Aurora, Colorado). Each of these electrodes was fed by a different stimulator. A common, much larger (10 × 10 cm) reference electrode was mounted over the right shoulder. We induced oscillatory currents at 55 Hz that were modulated by a 6 Hz envelope, to closely mimic the endogenous phase-amplitude modulation phenomenon occurring in the human cortex during cognitive tasks^27^. The maximum peak-to-peak current delivery by the stimulators was 2 mA (occurring at the points of maximum amplitude modulation). In the present study, no EEG measures during tACS were carried out because of technical difficulties in separating brain activity from the continuous alternating electric field induced by tACS (it may generally be possible to distinguish tACS artefacts from EEG data if the tACS electrodes are physically separated from the EEG electrodes^17^,^19^, but such a separation is not possible for our study where the tACS electrodes are necessarily mounted over the regions of interest for EEG recordings). In any case, recent studies in humans suggest that tACS is capable of entraining brain oscillations and modulating brain activity in a frequency- and topographic-specific manner^20^,^23^. Moreover, Ozen *et al.*^22^ showed with neural recordings acquired during tACS in rats that neocortical neurons oscillate in phase with the oscillatory electric field applied over the scalp, thus providing direct physiological evidence that tACS is capable of exogenously entraining cortical activity at the externally applied frequency. These empirical results have been more recently confirmed by computational modelling^21^ and empirical work^16^,^24^,^46^.

During pilot experiments we observed that when the subjects were engaged in the decision task, they could not discriminate in a given stimulation block (real or sham) what type of stimulation they were actually receiving. Moreover, subjects were unaware of the hypothesized effects of the stimulation on the behavioural task. Therefore, every 35 trials during the interleaved perceptual and value-based choices, the stimulation was switched on or off (Fig. 1b). Subjects were randomly assigned to participate either in experiment 1 or experiment 2 and were not aware (blinded) of the behavioural consequences of applying tACS.

In experiment 3, a new set of participants received tACS over the mFPC and parietal cortex at 6 different phases (or lags between the oscillations in the two regions) within the same experimental session: 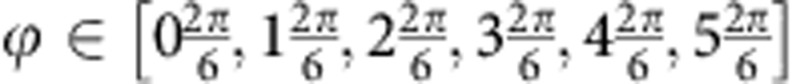 and sham. Every 25 trials the stimulation was randomly switched to one of these seven stimulation conditions. Behaviour from this experiment was modelled using a sinusoidal model written as a linear function of sines and cosines, that is, accuracy=*β*_0_+*β*_1_ sin(*ϕ*)+*β*_2_ cos(*ϕ*). The model was fitted using a Bayesian hierarchical framework based on a mixed-effects regression where all coefficient estimates were treated as random effects across the population.

### Behavioural analysis

To investigate the influence of tACS on the accuracy of responses for each experiment, we performed a hierarchical logistic mixed-effects regression of choices (correct=1, incorrect=0) on various regressors of interest, namely: task-relevant evidence level (1 to 4), stimulation (on=1, off=−1), task-irrelevant evidence level (that is, value-based for perceptual choices and perceptual for value-based choices, 1 to 4), hunger level (based on subject's hunger ratings collected before the beginning of the decision-making task, ranging from 1 to 5) and the interaction of task-relevant evidence level and stimulation. The mixed-effects regression had random effects for subject-specific constants and slopes. The results of these regressions are summarized in Supplementary Table 1. To investigate the influence of tACS on RTs, we performed a similar linear mixed-effects regression. We carried out the regressions with raw RTs and also log-transformed RTs (to improve normalization). None of these analyses revealed a significant influence of stimulation on RTs. No statistical methods were used to predetermine the sample size. Our choice of sample size was based on previous work using similar behavioural and transcranial stimulation protocols^13^,^16^.

### Computational model

We analysed the influence of tACS on value-based and perceptual choices with a prominent mathematical model of two-alternative decisions (the DDM)^18^ which incorporates both observed choices and reaction times to decompose the decision process into distinct latent variables corresponding to distinct aspects of the choice process^47^: (1) the efficiency of sensory evidence accumulation, known as the drift rate (*δ*); (2) any bias in the choice process (*β*); (3) the amount of evidence required to take a decision, known as the decision threshold (*α*); and (4) the delay in the onset of evidence accumulation, the non-decision time (*τ*). In addition, we included in this model two terms capturing the trial-to-trial variability of the drift rate (*η*) and the non-decision time (*χ*). This model therefore enabled us to directly study whether tACS had a specific influence on any of these parameters that influence the accuracy and speediness of the decisions.

The decision-making model implemented here is based on a simple one-dimensional Wiener process^48^: a dynamical system where the state of evidence *X*(*t*) at time *t* evolves via the stochastic equation 
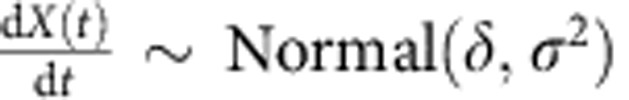
, where *δ* represents the quality of information processing defined as *δ=kE*, where *E* represents the evidence level (see equations (1) and (2) above) and *k* a variable that linearly scales the evidence (drift-rate). For initial conditions, where *β* represents an initial bias in the process, it is assumed that the system makes a decision ζ at time *t*_d_ whenever *X*(*t*)>=*α* (that is, a correct choice) or *X*(*t*)≤0 (that is, an incorrect choice). In addition, we accounted for visual processing and corticomuscular responses delays via the non-decision time parameter *τ* (the RT in each trial is defined as RT=*t*_d_+*τ*). The goal is to find the Wiener distribution Wiener(*δ*, *α*, *τ*, *β*) that best explains the distribution of empirical choices *y*(ζ, RT). To this end, we implement a hierarchical-Bayesian model where each individual data point *y*_(c,s,i)_(ζ, RT) follows a Wiener distribution^48^,^49^


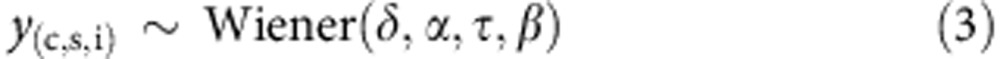


with indices *c* for conditions (*c*=*v* for value based, *c*=*p* for perceptual), *s* for subjects (*s*=1,...,*N*_subjects_) and *i* for trials (*i*=1,...*N*_trials_).

The hierarchical structure contains three levels of random variation: The trial, participant and condition. At the trial level, the non-decision time *τ* and the drift rate *δ* are assumed to vary trial-by-trial:


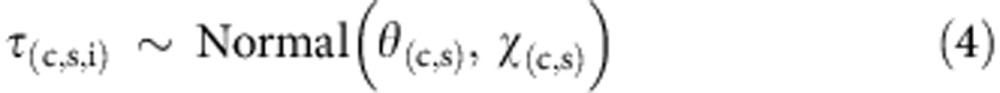



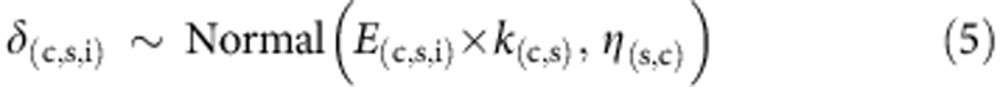


where Normal(*x*,*y*) represents a normal distribution with mean=*x* and s.d.=*y, E* represents the trial-by-trial evidence (see equations (1) and (2) above) and *k* is the drift-rate scale. Thus, *χ* and *η* represent the trial-to-trial variability associated with the non-decision times and the drift-rate.

Considering the biological plausibility of the model, decision thresholds should not include a variability term, as it is assumed that decisions are made once a predetermined threshold is crossed^50^. We therefore treated the boundary separation *α*_(c,s,i)_ as constant across trials (for a given participant) but treated all interindividual differences per stimulation condition level as random effects:


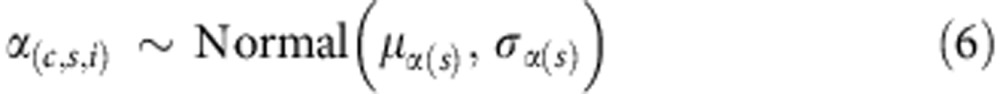


Given that we were working with absolute value differences, we assumed an unbiased diffusion process, that is, *β*_(c,s,i)_=0.5. Condition-specific population distributions (normal distributions) were also assumed for the trial-to-trial variability parameters of the drift rate (*η*) and of the non-decision time (*χ*), as this simplifies the comparison of these terms across experimental conditions. For latent variables at the highest level of the hierarchy (also known as hypergroup parameters), we assumed flat uninformed priors (that is, uniform distributions). The resulting hierarchical-Bayesian DDM is shown in Supplementary Fig. 3.

Posterior inference of the parameters in the hierarchical-Bayesian models was performed via the Gibbs sampler using the Markov Chain Montecarlo (MCMC) technique implemented in JAGS^51^. A total of 1,000,000 samples were drawn from an initial burn-in step and subsequently a total of new 1,000,000 samples were drawn with three chains (each chain was derived based on a different random number generator engine, and each with a different seed). We applied a thinning of 1,000 to this final sample, thus resulting in a final set of 1,000 samples for each parameter. This thinning assured that the final samples were autodecorrelated for all of the latent variables of interest investigated in this study. We conducted Gelman–Rubin tests^52^ for each parameter to confirm convergence of the chains. All latent variables in our Bayesian models had 
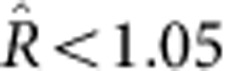
, which suggests that all three chains converged to a target posterior distribution. Posterior population distributions estimated for each parameter were compared (subtracted) between tACS conditions (on/off), and we tested whether the resulting distribution (effect) significantly differed from zero (that is, the null hypothesis) by means of the cumulative function up to/from 0 depending on the direction of the effect. We refer to this probability in the main text as *P*_MCMC_.

## Additional information

**How to cite this article:** Polanía, R. *et al.* The precision of value-based choices depends causally on fronto-parietal phase coupling. *Nat. Commun.* 6:8090 doi: 10.1038/ncomms9090 (2015).

## Supplementary Material

Supplementary Information Supplementary Figures 1-8 and Supplementary Tables 1-3
