# Supplementary material for: The precision of value-based choices depends causally on fronto-parietal phase coupling
Source: Nat Commun. 2015 Aug 20;6:8090. doi: 10.1038/ncomms9090 (PMC4560799; doi:10.1038/ncomms9090)
Supplement: Supplementary Information — Supplementary Figures 1-8 and Supplementary Tables 1-3 [file ncomms9090-s1.pdf]

SUPPLEMENTARY INFORMATION

The precision of value-based choices depends causally on fronto-parietal phase-coupling

Authors

Rafael Polanía[*1], Marius Moisa[*1], Alexander Opitz[2,3], Marcus Grueschow[1] & Christian C. Ruff[1]

(1) Laboratory for Social and Neural Systems Research (SNS-Lab)
Department of Economics
University of Zurich
8006 Zurich, Switzerland

(2) Nathan Kline Institute for Psychiatric Research
Orangeburg, New York, USA

(3) Center for the Developing Brain, Child Mind Institute
New York, New York, USA

(*) These two authors contributed equally to this work

Correspondence to:

- Rafael Polania, Department of Economics, University of Zurich, 8006 Zurich, Blümlisalpstrasse 10, Tel: +41-446345558,
  e-mail: rafael.polania@econ.uzh.ch

- Christian Ruff, Department of Economics, University of Zurich, 8006 Zurich, Blümlisalpstrasse 10, Tel: +41-446345067,
  e-mail: christian.ruff@econ.uzh.ch

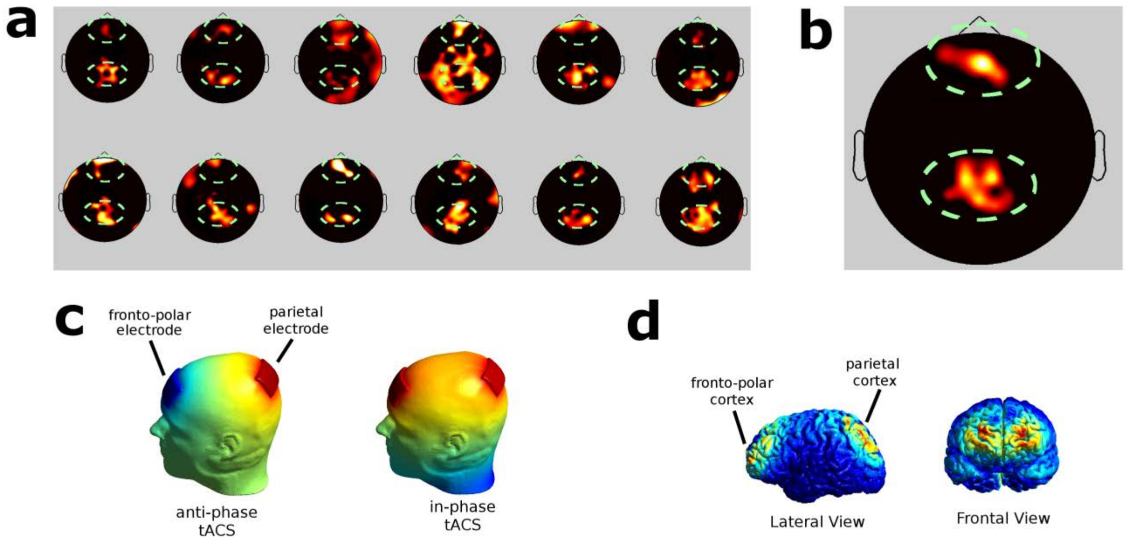

Supplementary Figure 1. Individual EEG scalp analysis and tACS electric field modelling. (a) Individual scalp maps of different subjects from the EEG study showing consistent parietal and fronto-polar activations with the group average statistics group results (shown in **b**). Green-dashed ellipses mark the region of interest in our study, namely the parietal and fronto-polar cortex. **(c)** Electrode montage in our two tACS conditions. Colours represent normalized current distribution over the scalp**. (d)** Normalized electric field distributions on the cortex resulting from our tACS electrode montage for the anti-phase condition using a realistic finite element head model. The in-phase condition produces a similar map. The maxima of the predicted electric field are relatively focal and occur precisely in our regions of interest: the posterior parietal lobule and the medial fronto-polar cortex. These are the very regions we intended to target, based on our scalp EEG data and previous MEG studies using a similar modelling approach during value-based choices[19].

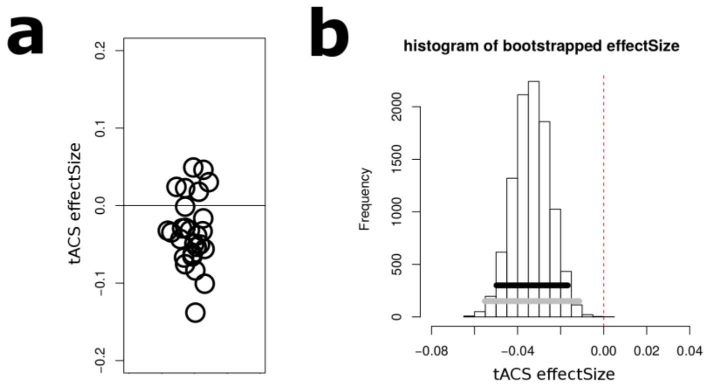

Supplementary Figure 2. tACS-induced main effects are robust across the population. (a) Effect size for each participant in the anti-phasic tACS effects on value-based choice accuracies. **(b)** Non-parametric boot-strapped population estimate of the effect-size (R=10,000 permutations). Thick black and grey lines at the bottom of the histogram show the 95% and 99% confidence intervals, respectively.

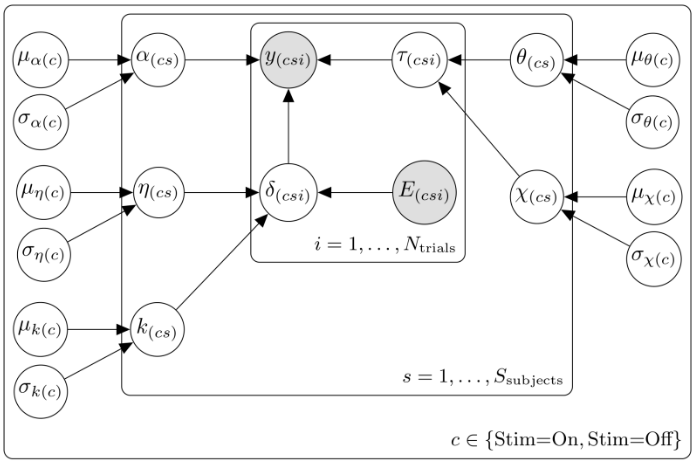

$$y_{(csi)} \sim \text{Wiener}(\alpha_{(cs)}, \beta, \tau_{(csi)}, \delta_{(csi)})$$
$$\tau_{(csi)} \sim \text{Norm}(\theta_{(cs)}, \chi^2_{(cs)})$$
$$\delta_{(csi)} \sim \text{Norm}(k_{(cs)} \times E_{(csi)}, \eta^2_{(cs)})$$

$$\alpha_{(cs)} \sim \text{Norm}(\mu_{\alpha(c)}, \sigma^2_{\alpha(c)})$$
$$\theta_{(cs)} \sim \text{Norm}(\mu_{\theta(c)}, \sigma^2_{\theta(c)})$$
$$k_{(cs)} \sim \text{Norm}(\mu_{k(c)}, \sigma^2_{k(c)})$$
$$\eta_{(cs)} \sim \Gamma(\mu^2_{\eta(c)}/\sigma^2_{\eta(c)}, \mu_{\eta(c)}/\sigma^2_{\eta(c)})$$
$$\chi_{(cs)} \sim \Gamma(\mu^2_{\chi(c)}/\sigma^2_{\chi(c)}, \mu_{\chi(c)}/\sigma^2_{\chi(c)})$$

$$E = \{V_{\text{best}} - V_{\text{worst}}\} \quad \text{or} \quad \{S_{\text{biggest}} - S_{\text{smallest}}\}$$

Supplementary Figure 3. Hierarchical Bayesian DDM.

Graphical Representation of the hierarchical Bayesian DDM fitted to the empirical data for each decision-making task. Clear shapes indicate latent variables and filled shapes observed variables (the observed variables were choice data y, which also contain reaction time information (see methods); and the evidence for each trial E). Equations on the right side of the figure show the distributions assumed for each of the latent variables in the model (see methods). For latent variables at the highest level of the hierarchy (also known as hyper-group parameters, i.e., variables represented by μ_x and σ_x), we assumed flat uninformed priors (i.e. uniform distributions: k ~ dunif(-2, 2), α ~ dunif(0.001, 3), θ ~ dunif(0.001, 3), η ~ dunif(0.001, 50), χ ~ dunif(0.001, 50)).

Summary of the key model parameters:

- α → The decision threshold
- θ → The non-decision time
- χ → The non-decision time trial-to-trial variability
- k → The drift-rate parameter scaling the evidence E
- η → The drift-rate trial-to-trial variability

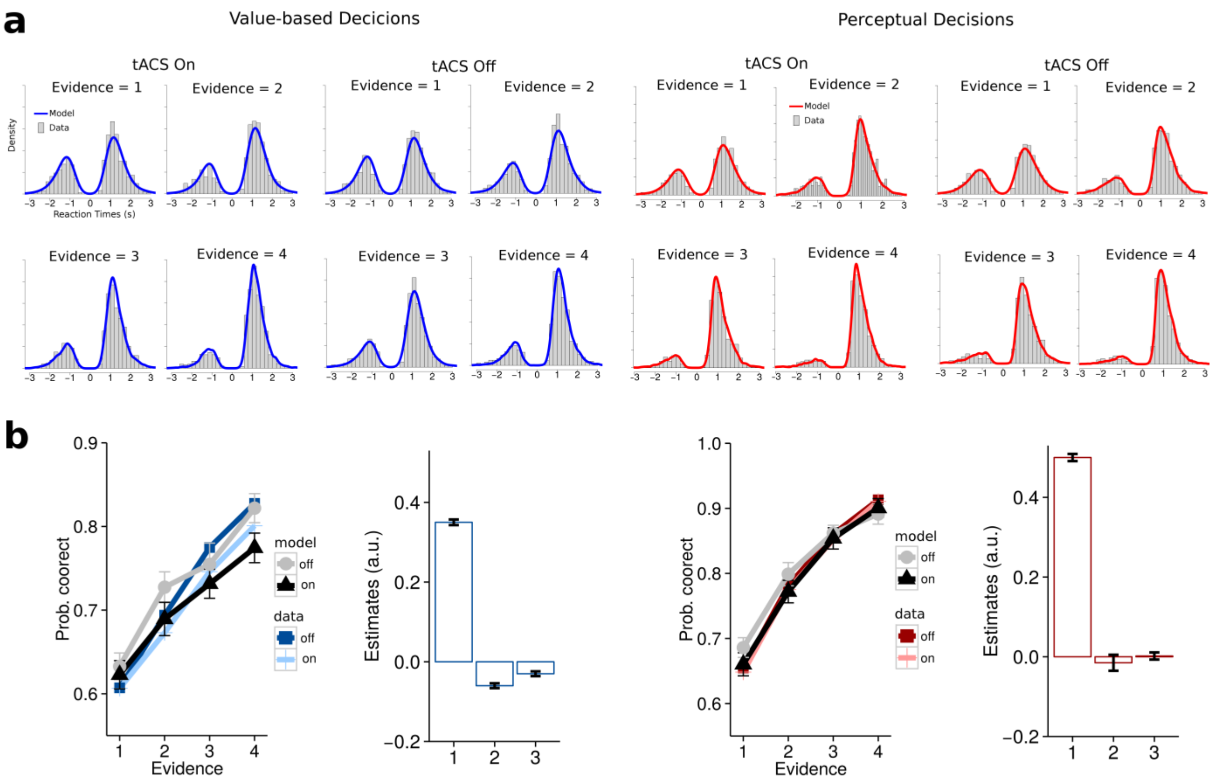

Supplementary Figure 4. The fitted DDM accurately models the empirical data. **(a)** Model predictions after fitting the empirical data to the DDM. Figures in this panel represent the reaction times (RT) distributions for correct (positive values on x-axis) and incorrect (negative values on x-axis) choices. Histograms represent the observed distributions of RT data and the overlaid lines represent the model predicted distributions. Data are shown for each choice type (value-based or perceptual choices) and each stimulation condition in the anti-phasic tACS stimulation protocol (experiment 1). **(b)** Model predictions of choice accuracy highly resemble the empirical observations. The model predicts that anti-phasic tACS produces more inaccurate value-based choices specifically for strong evidence levels (i.e., an evidence*stimulation interaction), but leaves perceptual choices unaffected (see also Figure 2 in main text).

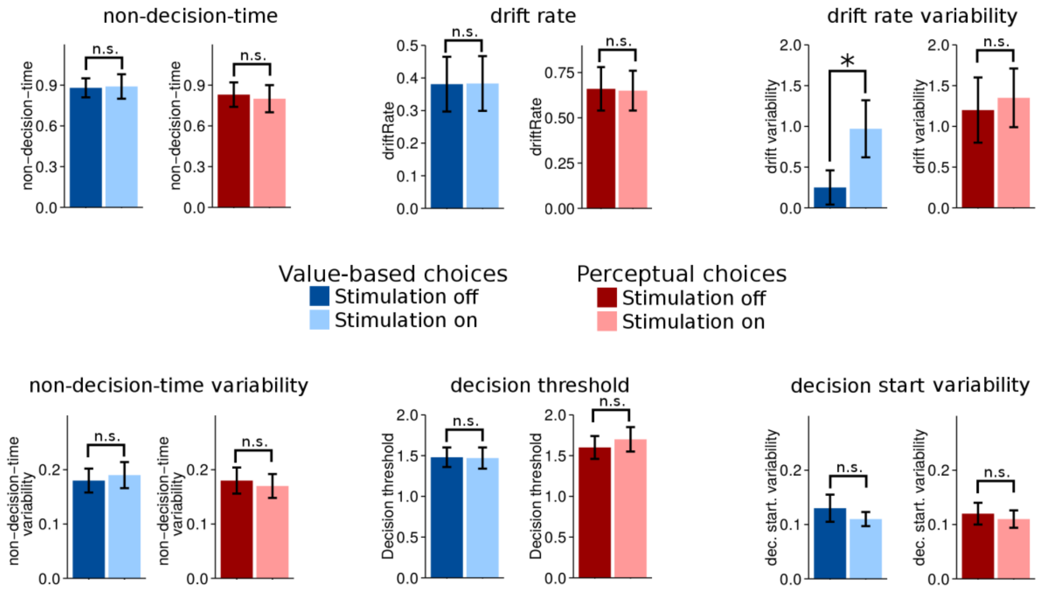

Supplementary Figure 5. tACS-induced trial-to-trial variability effects are robust to the inclusion of a decision start variability term in the model.

Estimation of model parameters fitted to our empirical data, this time including a decision start variability term (bottom-right panel) to the model fits. Our results remain unchanged, as the only model parameter that is affected by tACS is the drift-rate variability (top-right panel).

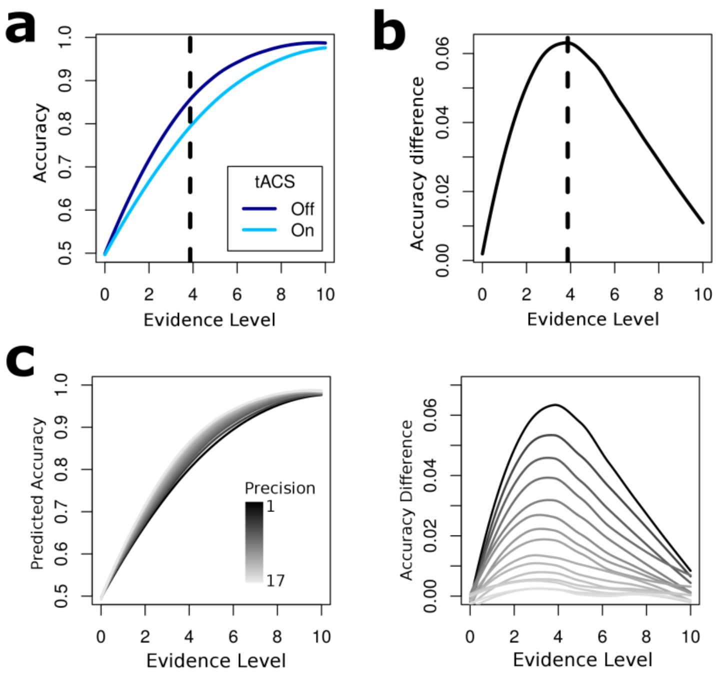

Supplementary Figure 6. tACS-induced changes in trial-to-trial variability explains stimulation x evidence interaction.

(a) Predicted accuracies of the DDM (with parameters fitted to our empirical data) for a wide range of evidence levels. **(b)** Predicted accuracy difference between tACS and no tACS. The vertical dashed line shows the evidence level with maximum accuracy difference, which corresponds to the maximum evidence level chosen for our study. **(c)** Predicted accuracies (left) and accuracy differences (right) for different levels of drift rate variability (different grey levels; note that variability is reciprocal to precision). Note that an increase in drift rate variability (as empirically observed during tACS) predicts the exact pattern of decreased accuracy for higher evidence levels as evident in our empirical data. These predictions were based solely on model fits to the baseline condition and not the stimulation condition, therefore addressing any concerns regarding model over-fitting.

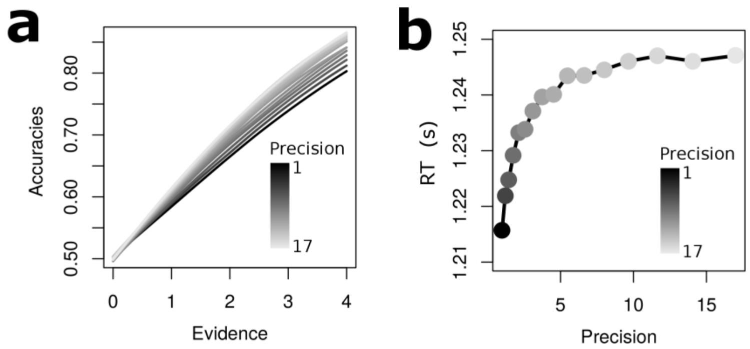

Supplementary Figure 7. Increased trial-to-trial variability leads to decreased accuracies and a slight decrease in RTs.

(a) Model-predicted accuracies as a function of different evidence levels (x-axis) and different drift-rate variability levels (grey colour scale; note that variability is reciprocal to precision). **(b)** Average reaction times (RT) as a function of different drift rate variability levels (grey colour scale). Note that increases in drift rate variability lead to the same pattern we observe in our data i.e. decreased accuracy and slightly faster RTs.

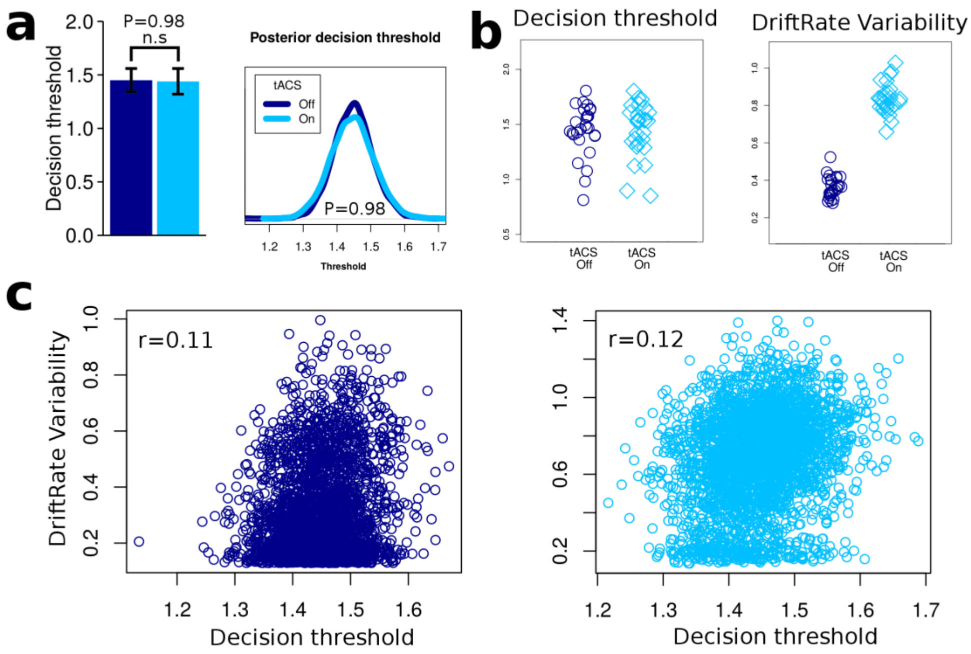

Supplementary Figure 8. Decision threshold and trial-to-trial variability are uncorrelated during model fits.

we show that the tACS effect on the decision boundary is virtually equal to zero. The probability that the null-hypothesis is accepted is P=0.98 (panel **a**). This confirms that there is no trend towards lower decision boundaries. Moreover, the distributions of this parameter are identical across tACS groups (panel **b**, left), in contrast to the distributions of the drift rate variability (panel **b**, right). Additionally, we ascertained that the decision threshold and the drift-rate variability are not correlated in the parameter estimation during model fits, by assessing the relationship of the Markov chain montecarlo (mcmc) samples. This analysis shows that the chains are de-correlated, therefore suggesting independence during model parameter estimation (panel **c**). All these analyses confirm that the decision boundary does not change, and that the reaction time effects in our data are fully consistent with the tACS effects of drift rate variability.

Value-based choices

Factor	Estimate	std. err	P-value
Evidence	0.33	0.051	<0.001
**tACS**	**-0.133**	**0.05**	**0.009**
Non-relevant evidence	0.02	0.026	0.94
hunger	-0.04	0.053	0.4
**Evidence * tACS**	**-0.1**	**0.05**	**0.035**

Perceptual choices

Factor	Estimate	std. err	P-value
Evidence	0.5	0.056	<0.001
tACS	-0.06	0.07	0.32
Non-relevant evidence	-0.01	0.03	0.8
hunger	0.02	0.05	0.62
Evidence * tACS	0.06	0.07	0.25

Supplementary Table 1. Logistic-mixed effects regression in experiment 1.

The influence of tACS on the accuracy of responses was initially investigated by means of a logistic mixed-effects regression of choices (correct=1, incorrect=0) on various regressors of interest, namely: task-relevant evidence level, stimulation (on=1, off=-1), task-irrelevant evidence (i.e., value-based for perceptual choices and perceptual for value-based choices), hunger level (based on subject's hunger ratings collected before the begin of the decision-making task) and the task-relevant evidence*stimulation interaction. The mixed effects regression had random effects for subject-specific constants and slopes. As expected, stronger evidence led to more correct choices for both types of task (main effect evidence). The stimulation (tACS) led to a significant negative main effect on value-based choice accuracy, and also to an evidence*stimulation interaction.

Value-based choices

Factor	Estimate	std. err	P-value
Evidence	0.39	0.045	<0.001
tACS	-0.05	0.061	0.38
Non-relevant evidence	-0.05	0.027	0.1
hunger	-0.04	0.06	0.5
Evidence * tACS	-0.03	0.055	0.56

Perceptual choices

Factor	Estimate	std. err	P-value
Evidence	0.6	0.07	<0.001
tACS	-0.01	0.08	0.81
Non-relevant evidence	0.008	0.03	0.77
hunger	-0.05	0.05	0.28
Evidence * tACS	-0.07	0.07	0.2

Supplementary Table 2. Logistic-mixed effects regression in experiment 2. Same as Supplementary Table 1, but this time for the group of subjects participating in the in-phase tACS experiment. Again, evidence level strongly affected choice accuracy for both types of decisions. However, this stimulation protocol did not have any significant influence on perceptual or value-based choices accuracy.

Accuracies

Factor	Estimate (β_i)	std. err	P-value
β_0	0.88	0.0025	<0.001
$\beta_1 \sin(\varphi)$	0.004	0.003	0.17
$\beta_2 \cos(\varphi)$	**0.009**	**0.003**	**0.018**

Supplementary Table 3. Hierarchical circular model regression in Experiment 3. The influence of tACS on the accuracy and RTs of responses was investigated by means of a hierarchical mixed effects circular regression (see main text). The mixed effects regression had random effects for all subject-specific factors. tACS influenced accuracies in a sinusoidal manner. The *cos(x)* component of the regression was significantly influenced. This is what we expected given that we hypothesized that accuracies should be higher when $\varphi \to 0$ (i.e. when $\cos(\varphi) \to 1$) and lowest when $\varphi \to \pi$ (i.e. when $\cos(\varphi) \to 0$). Please see Figure 4 in the main text.
